# *Granulicatella adiacens* prosthetic hip joint infection after dental treatment

**DOI:** 10.1099/jmmcr.0.005044

**Published:** 2016-06-25

**Authors:** Osama Aweid, Sabapathy Sundararajan, Abraham Teferi

**Affiliations:** ^1^​ Department of Trauma and Orthopaedics, Peterborough City Hospital, Edith Cavell Campus, Bretton Gate, Peterborough, Cambridgeshire, PE3 9GZ, UK; ^2^​ Department of Trauma and Orthopaedics, Luton and Dunstable University Hospital NHS Trust, Luton, LU4 0DZ, UK; ^3^​ Department of Microbiology, Luton and Dunstable University Hospital NHS Trust, Luton, LU4 0DZ, UK

**Keywords:** Treatment: antibiotics, Disease/Indication: prosthetic joint infection, Pathology/Symptoms: *Granulicatella adiacens*

## Abstract

**Introduction:**

*Granulicatella adiacens* is a Gram-positive bacteria and a normal component of oral flora. It is also found in dental plaques, endodontic abscesses and can rarely cause more serious infections.

**Case presentation:**

We describe a prosthetic hip joint infection in an 81-year-old fit and healthy man due to *Granulicatella adiacens* who underwent a prolonged dental intervention two days earlier without antibiotic prophylaxis. The infection was successfully treated with surgical intervention and a combination of antibiotics. The patient eventually succumbed to severe community-acquired pneumonia two months later.

**Conclusion:**

Current guidelines recommend avoidance of antibiotic prophylaxis prior to dental treatment in patients who have no co-morbidities and no prior operation on the index prosthetic joint. This case report indicates that infections of prosthetic joints may be associated with dental procedures even in fit and healthy patients without the recognized risk factors.

## Introduction

Species of the genus *Granulicatella* are a normal component of oral flora. They are also found in dental plaques and endodontic infections (Siqueira & Rôças, 2006). They can cause more serious infections and have mostly been identified in infective endocarditis with up to 17 cases noted in a recent review (Cargill *et al.,* 2012). Other reported infections include an abdominal aortic graft, Tesio haemodialysis catheter, vertebral osteomyelitis, native joint septic arthritis, discitis, peritoneal-dialysis-related peritonitis, and meningitis following neurosurgery (Cargill *et al.,* 2012). To date, only two cases of prosthetic joint infections due to *Granulicatella adiacens* have been published in the literature. The first report is a prosthetic knee joint infection in a 55-year-old diabetic man, one month after dental extraction (Mougari *et al.,* 2013). The second is prosthetic knee arthritis in a 43-year-old male with no medical co-morbidities or recent dental treatment (Riede *et al.,* 2004). His past surgical history prior to his total knee replacement did however include an open reconstruction of the anterior cruciate ligament and internal fixation of the patella with subsequent metal removal for a skiing injury, as well as six arthroscopies with the last being complicated by septic arthritis due to *Staphylococcus aureus*, *Escherichia coli* and enterococci.

In this case report, we describe a prosthetic hip joint infection due to *Granulicatella adiacens* occurring within two days after a dental intervention in a gentleman with no medical co-morbidities or prior septic arthritis.

## Case report

**Fig. 1.**
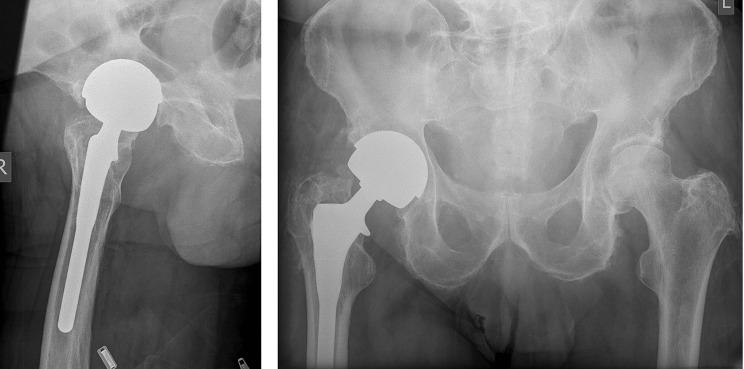
Anteroposterior (AP) and lateral radiograph of the right hip showing a Furlong uncemented metal-on-metal hip replacement with no radiological signs of loosening or osteolysis.

An 81-year-old fit and healthy gentleman presented with severe right-sided hip pain on 22 January 2015. He had an uncemented right metal-on-metal hip replacement carried out in 2008 (see Fig. 1). At presentation he denied any relevant past medical history. He was not on any regular medications. He drinks 5–10 units of alcohol a week and does not smoke. Two days prior to his admission, he had a prolonged dental root canal treatment for a pulp infection at his dental surgery without antibiotic prophylaxis. His oral health was otherwise good. No cultures were taken at the time to confirm the presence or absence of *Granulicatella adiacens* in the oral cavity.

**Fig. 2.**
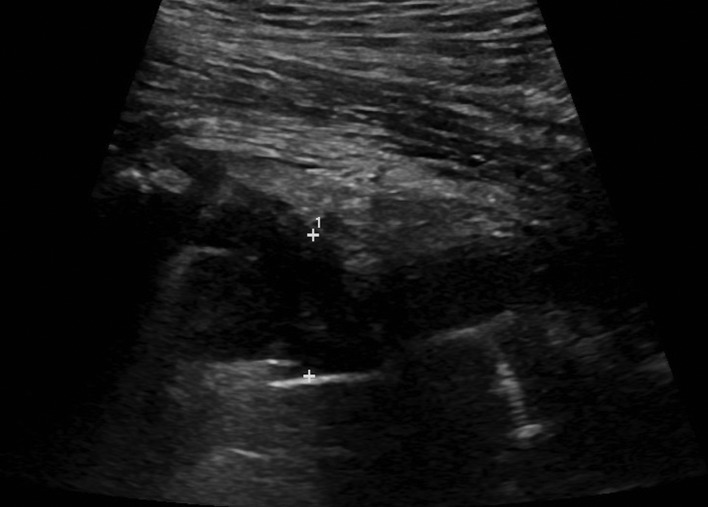
Ultrasound scan of the right hip showing a hip joint collection with a depth of 18 mm.

On physical examination the only pertinent physical findings were that he had a temperature of 38.5 °C with a swollen and erythematous right hip joint. His blood results on admission showed C-reactive protein (CRP) 12.9 mg l^−1^, white blood cells 17.6×10^9^ l^−1^, and neutrophils 16.11×10^9^ l^−1^. A blood culture taken on the day of admission did not grow any organism. An ultrasound-guided aspirate of his right hip (see Fig. 2) revealed thick purulent fluid. Empirically benzylpenicillin (1 g every 6 h) and flucloxacillin (2 g every 6 h) were started. The direct Gram stain showed many pus cells and Gram-positive cocci in chains. The isolate did not grow on blood and chocolate agar, but did grow on anaerobic plates and was identified as *Granulicatella adiacens* by matrix-assisted laser desorption/ionization time-of-flight mass spectrometry (MALDI-TOF MS) (Bruker) with a score value of 2.185. Antibiotic sensitivities were analysed by the disc diffusion method according to the British Society for Antimicrobial Chemotherapy (BSAC) guidelines and yielded the following sensitivities: erythromycin (resistant), vancomycin (sensitive) and penicillin (sensitive). There was no co-infection with *Staphylococcus aureus*.

On the fourth day of admission, an open washout and debridement of the right hip was undertaken. Pus was noted within the joint with some tracking at the metal–bone interface of the stem anteriorly and proximally. However, the stem was very well fixed with no loosening. Two tissue specimens were sent for culture and no organism was isolated after a long incubation. As the organism was isolated from only the initial aspirate specimen, the antibiotics were changed to a regimen with wide coverage: intravenous vancomycin (500 mg every 12 h) and piperacillin with tazobactam (4.5 g every 8 h), and oral fucidic acid (500 mg every 8 h).

The inflammatory markers showed a steady decline and the patient improved symptomatically. He was then sent home and prescribed six weeks of outpatient antimicrobial therapy (OPAT) but he was re-admitted after a month for an urticarial rash due to an adverse drug reaction. In addition, he also had several temperature spikes and therefore underwent a second surgical exploration and washout the following day and another five days later. Intra-operatively the soft tissues and joint showed no evidence of infection, no organism was seen on Gram stain and no microorganism was isolated from the tissue samples on both occasions. Considering the adverse drug reaction, the antibiotic regimen was changed to daptomycin (60 mg once daily) and meropenem (2 g every 8 h) and used to complete his six-week course of IV antibiotics. His second time hospitalization lasted a period of 10 days in total.

The patient made a good recovery with a resolving rash, a C-reactive protein of 18 mg l^−1^, a normal white cell count, and a fully healed wound. He was referred to a regional Bone Infection Unit for consideration of a two-stage revision hip replacement. Seven weeks following his second hospital discharge and prior to his revision surgery, he was admitted with cough, shortness of breath and raised inflammatory markers (C-reactive protein 312 mg l^−1^, white cell count 22×10^9^ l^−1^). He was diagnosed with severe community-acquired pneumonia and succumbed to the infection three days later. The pathogen that caused the community pneumonia was not identified.

## Discussion

Members of the genus *Granulicatella* are catalase-negative, facultatively anaerobic, Gram-positive cocci, and currently there are three described species of the genus: *Granulicatella adiacens*, *Granulicatella elegans* and *Granulicatella balaenopterae* (Collins & Lawson, 2000). They belong to the family *Carnobacteriaceae* and are uncommon clinical isolates (Cargill *et al.,* 2012).

*Granulicatella adiacens* causing a prosthetic joint infection has previously been reported in a 55-year-old diabetic male (Mougari *et al.,* 2013) and in a 43-year-old male with a known history of prior septic arthritis and multiple surgical interventions to the same joint (Riede *et al.,* 2004). To the best of our knowledge, our case is the first report of a *G. adiacens* prosthetic joint infection following dental treatment in a patient with no medical co-morbidities or previous joint infections. Although *G. adiacens* was not isolated by oral sampling or identified in blood culture, the occurrence of the prosthetic infection with an organism normally found in oral flora so soon after a dental procedure suggests that the most likely source of the patient's infection would have been blood-borne dissemination of oral flora following the root canal treatment. Infections associated with prosthetic joints represent the most devastating implant-associated complication with high morbidity, substantial cost and often lead to a protracted hospitalization (Trampuz & Zimmerli, 2005). Administering antibiotic prophylaxis during dental treatment for patients with joint prosthetics to reduce the risk of prosthetic infection is however, controversial.

The American Dental Association (ADA) and the American Academy of Orthopaedic Surgeons (AAOS) initially recommended antibiotic prophylaxis prior to any dental procedure in all patients with prosthetic joints regardless of medical co-morbidities (American Academy of Orthopaedic Surgeons, 2009). In December 2012, the AAOS and ADA released a new clinical practice guideline and, based on a systematic review of the literature, were unable to recommend for or against the use of oral antimicrobials in any patients with prosthetic joint implants undergoing dental procedures. The guideline stated ‘*the patient should have an important role and ultimately make the decision to discontinue antibiotics before a dental procedure*’ (Watters *et al.,* 2013). The review however failed to identify patient characteristics and while collaborative decision-making between patients and clinicians is important, there was concern over the emphasis on patients’ judgement in making the clinical decision (Young *et al.,* 2014). As a result, the release of this guideline was followed by calls to the ADA for additional clarification. In 2014, the ADA released an updated and more specific set of clinical practice guidelines which took into account four case-control studies and concluded that ‘*prophylactic antibiotics are not recommended prior to dental procedures to prevent prosthetic joint infection. The practitioner and patient should consider possible clinical circumstances (such as the presence of immune-compromise, previous periprosthetic joint infection, poor nutrition, haemophilia, or a malignancy) that may suggest the presence of a significant medical risk in providing dental care without antibiotic prophylaxis, as well as the known risks of frequent or widespread antibiotic use*’ (Sollecito *et al.,* 2015). This position is supported by a recent Journal of Bone and Joint Surgery (JBJS) current concepts systematic review (Young *et al.,* 2014) and is similar to that taken by the British Society for Antimicrobial Chemotherapy (Simmons *et al.,* 1992).

In our report, a prosthetic hip joint infection with a bacterium found in oral flora occurred very soon after a root canal treatment suggesting that the infection may be directly linked to the dental treatment. Current guidelines recommend avoidance of antibiotic prophylaxis prior to dental procedures (like our patient) who have no co-morbidities and no prior operation on the index joint or post-operative complications. This case illustrates that bacteraemia due to dental treatment can be risky even in fit and healthy patients without the recognized risk factors for prosthetic joint infection. Although a single case may not be enough to change antibiotic prophylaxis guidelines, we believe that the benefits of administration of one dose of broad-spectrum antibiotic (based on local microbial prevalence) immediately prior to dental intervention in all patients with prosthetic joints, including those with no medical co-morbidities or prior surgery, may outweigh the risk of antibiotic-related adverse effects and potential drug resistance particularly if further such cases are reported in the literature.
